# Cerebrospinal fluid soluble TREM2 in aging and Alzheimer’s disease

**DOI:** 10.1186/s13195-016-0182-1

**Published:** 2016-04-27

**Authors:** Kristi Henjum, Ina S. Almdahl, Vibeke Årskog, Lennart Minthon, Oskar Hansson, Tormod Fladby, Lars N. G. Nilsson

**Affiliations:** Department of Pharmacology, University of Oslo and Oslo University Hospital, P.b. 1057, Blindern NO-0316, Oslo, Norway; Department of Neurology, Akershus University Hospital, Lørenskog, Norway; Department of Neurology, Faculty Division, Akershus University Hospital, University of Oslo, Lørenskog, Norway; Department of Clinical Sciences Malmö, Memory Clinic, Clinical Memory Research Unit, Lund University, Malmö, Sweden

**Keywords:** Aging, Alzheimer’s disease, Amyloid beta, Microgliosis, Mild cognitive impairment, Neuroinflammation, Soluble TREM2, Tau

## Abstract

**Background:**

Alzheimer’s disease (AD) neuropathology is associated with neuroinflammation, but there are few useful biomarkers. Mutant variants of triggering receptor expressed on myeloid cells 2 (TREM2) have recently been linked to late-onset AD and other neurodegenerative disorders. TREM2, a microglial receptor, is involved in innate immunity. A cleaved fragment, soluble TREM2 (sTREM2), is present in the cerebrospinal fluid (CSF).

**Methods:**

We developed and used a novel enzyme-linked immunosorbent assay to investigate the potential value of CSF sTREM2 as an AD biomarker in two independent cohorts: an AD/mild cognitive impairment (MCI)/control cohort (*n* = 100) and an AD/control cohort (*n* = 50).

**Results:**

We found no significant difference in sTREM2 levels between groups of controls and patients with AD or MCI. However, among all controls there was a positive correlation between sTREM2 and age (Spearman rho = 0.50; *p* < 0.001; *n* = 75). In the AD/MCI/control cohort, CSF sTREM2 correlated positively with total Tau (T-tau) (Spearman rho 0.57; *p* < 0.001; *n* = 50), phosphorylated Tau (P-tau) (Spearman rho 0.63; *p* < 0.001; *n* = 50) and amyloid-β1–42 (Aβ42) (Spearman rho 0.35; *p* = 0.01; *n* = 50) in control subjects. Among controls with a CSF Aβ42 above a cut-off value (700 pg/ml) in this cohort, the positive correlation between sTREM2 and Aβ42 was stronger (Spearman rho = 0.44; *p* = 0.002; *n* = 46).

**Conclusions:**

sTREM2 in CSF correlates with aging in controls, and with the neurodegenerative markers CSF T-tau/P-tau among controls who are negative for AD CSF core biomarkers Aβ42, T-tau or P-tau.

**Electronic supplementary material:**

The online version of this article (doi:10.1186/s13195-016-0182-1) contains supplementary material, which is available to authorized users.

## Background

Alzheimer’s disease (AD) is characterized by neurodegeneration in the presence of two neuropathological hallmarks, extracellular amyloid-beta (Aβ) plaques and intracellular neurofibrillary tangles (NFTs), in certain brain regions [1]. A definitive AD diagnosis relies on post-mortem histological confirmation, but imaging and analyses of cerebrospinal fluid (CSF) Aβ1–42 (Aβ42), total Tau (T-tau) and phosphorylated Tau (P-tau) have transformed the field by enabling early and reliable ante-mortem AD diagnosis. These biomarkers are now included in the diagnostic guidelines [2–4], but additional AD biomarkers are needed to measure specific aspects and progression of this complex disease. The AD brain is also affected by neuroinflammation, but the immune reactions involved are intricate and their role in pathogenesis is partly unclear. Moreover biomarkers of neuroinflammation are scarce [5]. Longitudinal monitoring of astroglial or microglial activation with positron emission tomography (PET) imaging is used in research but not established in clinical practice [6]. In CSF, the potential value of many immune mediators as biomarkers has been examined. A diagnostic or prognostic value of different complement factors, acute-phase proteins, cytokines or chemokines has been reported, but findings have seldom been robust and reproducible [7]. YKL-40 might become a clinically useful marker of astrogliosis [8], but markers of microgliosis will also be needed.

Multiple genetic studies of late-onset AD point to a causative role of innate immunity in the AD pathogenesis [9–11]. Triggering receptor expressed on myeloid cells 2 (TREM2) is expressed abundantly by microglia [12, 13] and *TREM2* is a susceptibility gene for late-onset AD [14, 15]. Gene-expression analyses of late-onset AD post-mortem brain also suggest that an immune-specific and microglia-specific module around the TREM2 signalling adapter DNAX activating protein 12 (DAP12) is involved in the pathogenesis [16]. Moreover, TREM2 expression is elevated with aging in human brain [12] and in the vicinity of amyloid deposits in transgenic mouse models of AD [17]. TREM2 is a transmembrane innate immune receptor undergoing ectodomain cleavage with extracellular release of a soluble TREM2 (sTREM2) fragment which is detectable in CSF [18, 19]. A disintegrin and metalloproteinase (ADAM)-10, a key enzyme for α-secretase cleavage of Aβ precursor protein (AβPP), cleaves the TREM2 ectodomain [18]. The remaining TREM2 C-terminal fragment is digested by γ-secretase [20]. Since both genetic and pathological studies link *TREM2* to AD, sTREM2 might be a useful biomarker of microglial activation or neurodegeneration. Improved abilities to monitor microglial function and activity would also facilitate development of new microglial-based therapeutics. In the present study, we developed and validated an enzyme-linked immunosorbent assay (ELISA) and explored whether sTREM2 could serve as a diagnostic biomarker for AD or mild cognitive impairment (MCI). Moreover, we examined whether sTREM2 levels correlated with the established AD CSF core biomarkers Aβ42, T-tau or P-tau. We also analysed the effect of normal aging, the most important risk factor of AD.

## Methods

### Clinical samples

The Swedish cohort was from the Memory Clinic of Skåne University Hospital in Malmö, Sweden, and comprised 25 patients diagnosed with AD and 25 non-AD individuals (controls). Patients diagnosed with AD met the DSM-IIIR criteria for dementia [21] and the criteria for probable AD, as defined by the National Institute of Neurological and Communicative Disorders and Stroke (NINCDS-ADRDA) [22]. All subjects were carefully assessed and tested by medical doctors with extensive experience in cognitive disorders. Their brains were examined with either magnetic resonance imaging (MRI) or computed tomography (CT). Controls were clinically followed up to ensure that the cognitive complaints at baseline were not due to dementia or any other neurodegenerative disorder. The CSF samples of all patients were collected as part of routine clinical investigation. In conjunction with the investigation, oral informed consent for future use of their banked CSF samples for research purposes was obtained and documented in the patients’ medical records. All patients were later instructed to withdraw their permission if they changed their minds, as advertised in the local press. The design of the study was approved by the Local Ethics Committee of Lund University in Sweden (permit 2010-401), and the study procedure was conducted in accordance with the Declaration of Helsinki.

The Norwegian cohort was from the Memory Clinic of Akershus University Hospital in Lørenskog, Norway. The cohort encompassed 50 patients diagnosed with either AD or MCI, due to a pre-dementia stage of AD, and 50 cognitively healthy controls. All patients were interviewed and examined by a physician trained in diagnosing cognitive disorders. They all underwent cognitive testing, either cerebral MRI or CT, blood screening and standard lumbar puncture as part of the clinical assessment. Patients met either the National Institute on Aging–Alzheimer’s Association (NIA-AA) criteria for dementia due to AD [4] or the high-likelihood NIA-AA criteria for MCI due to AD [2] (29 patients and 21 patients, respectively). The controls were either orthopaedic patients scheduled for elective joint replacement surgery, spouses of patients attending the Memory Clinic or individuals recruited through newspaper advertisement. CSF was collected before administration of spinal anaesthesia in the orthopaedic patients. The remaining controls underwent standard lumbar puncture. Inclusion criteria for the controls were the absence of any reported cognitive complaints and normal CSF Aβ42 concentrations according to the cut-off value set by the laboratory (>550 pg/ml, modified from [23, 24]). All controls were invited to undergo further assessments; 35 consented to cognitive testing and 32 to cerebral MRI. Exclusion criteria for both AD patients and controls were: any ongoing severe neurological, medical or psychiatric co-morbidity or treatment with the potential to impair cognitive functioning; systemic inflammatory disease or infection based on clinical and laboratory assessment; and use of immunosuppressant drugs. The three groups were matched for age and gender. The Regional Committee for Medical and Health Research Ethics, South East Norway, approved the study (approval 1.2007.2511, 2011/1015 and 2013/150) and all participants gave written informed consent. The study procedure was conducted in accordance with the Declaration of Helsinki. The demographics and clinical characteristics of both cohorts are presented in Table 1.

**Table 1:** Characteristics of the Norwegian and Swedish cohorts

				*p* value		
	Controls	MCI	AD	Controls–MCI	Controls–AD	MCI–AD
Norwegian cohort	*n* = 50	*n* = 21	*n* = 29			
Gender						
Women	25	12	13			
Men	25	9	16			
Age	66 (50–86)	67 (55–75)	68 (56–75)			
MMSE	29 (29–30)	27 (26–29)	20 (17–24)	*	*	*
CSF Aβ42 (pg/ml)	1010 (880–1188)	494 (356–531)	500 (386–553)	*	*	0.43
CSF T-tau (pg/ml)	307 (201–391)	628 (497–927)	772 (647–1143)	*	*	0.06
CSF P-tau (pg/ml)	51 (38–61)	75 (62–111)	72 (67–91)	*	*	1.00
ApoE genotype						
E2/E3	8	–	1			
E2/E4	–		1			
E3/E3	35	5	5			
E3/E4	6	8	12			
E4/E4	–	8	10			
Not known	1					
CSF sTREM2 (ng/ml)	4.4 (3.0–5.7)	4.1 (2.4–5.9)	4.8 (3.5–7.1)	0.42	0.17	0.11
Swedish cohort	*n* = 25		*n* = 25			
Gender						
Women	17		18			
Men	8		7			
Age	62 (43–80)		79 (61–86)			
MMSE	29 (28–30)		18 (13–22)		*	
CSF Aβ42 (pg/ml)	520 (469–597)		340 (265–430)		*	
CSF T-tau (pg/ml)	380 (233–480)		670 (490–895)		*	
CSF P-tau (pg/ml)	44 (36–61)		80 (69–96)		*	
ApoE genotype						
E2/E3	5		1			
E2/E4	1		1			
E3/E3	14		4			
E3/E4	4		15			
E4/E4	1		4			
CSF sTREM2 (ng/ml)	3.2 (2.8–5.0)		3.8 (2.6–5.6)		0.76	

Descriptive statistics of gender, age, MMSE, ApoE genotype and CSF biomarker data of AD, MCI and control patients of the two cohorts

Data are presented as median (interquartile range), except age which is presented as median (minimum–maximum). Two-tailed *p* values obtained by Mann–Whitney *U* test for group comparison

*p < 0.001

*Aβ42* amyloid beta 1–42, *AD* Alzheimer’s disease, *ApoE* apolipoprotein E, *CSF* cerebrospinal fluid, *MCI* mild cognitive impairment, *MMSE* Mini-Mental State Examination, *P-tau* phosphorylated Tau, *sTREM2* soluble triggering receptor expressed on myeloid cells 2, *T-tau* total Tau

The TREM2 [p. T66M] mutation prevents shedding of TREM2, and CSF from a patient homozygous for [p. T66M] is reported to be devoid of sTREM2 [18]. A sample from such a patient was used to verify that the ELISA signal was specific for sTREM2, and not partly due to other components in the CSF matrix. Genomic DNA from the [p. T66M] mutation carrier had been analysed by exome sequencing of the entire genome [25].

### CSF collection and storage

Lumbar puncture was in general performed between 9 a.m. and 12 p.m., predominantly in the L3/L4 or L4/L5 inter-space and without any reported serious adverse effects. CSF was collected in polypropylene tubes, centrifuged and stored at −80 °C. The samples were subjected to a maximum of two freeze–thaw cycles prior to determination of sTREM2 levels.

### Cell cultures

The Chinese hamster ovary CHO-K1 and THP-1 cell lines were purchased from American Type Culture Collection (ATCC, Rockville, MD, USA). CHO-K1 cells were cultured in Dulbecco’s modified Eagle medium (DMEM) supplemented with 10 % fetal bovine serum (FBS) and 1 % penicillin–streptomycin (Sigma, St. Louis, MO, USA). These cells were transfected with a synthetic human TREM2-DAP12 fusion gene that had been subcloned into the *Nhe*I and *Bg*III sites and thus replaced copGFP in a pmaxGFP expression vector (Lonza, Basel, Switzerland). Transfections were carried out using Lipofectamine LTX and Plus Reagent (Life Technologies, Carlsbad, CA, USA) according to the manufacturer’s recommendations. Conditioned cell culture medium (conditioned medium) was collected 15 h after transfection, centrifuged at 5200 × *g* for 15 min at 4 °C and supplemented with Complete® protease inhibitor (Roche, Basel, Switzerland). The cells were washed with PBS and lysed with 0.5 % (w/v) sodium dodecyl sulphate (SDS) (Sigma) in PBS with Complete® and stored at –20 °C. The THP-1 monocyte cell line was cultured in RMPI with Glutamax (Thermo-Scientific, Waltham, MA, USA) supplemented with 10 % FBS and 1 % penicillin–streptomycin. For differentiation, 0.1 μM phorbol 12-myristate 13-acetate (Sigma) was added to the culture media. The conditioned medium from the differentiated cells was collected after 48 h and centrifuged at 5200 × *g* for 7 min. The supernatant was supplemented with Complete®, stored at –80 °C and used as an internal standard in the TREM2 ELISA.

### TREM2 ELISA

Maxisorp plates (ThermoFischer, Waltham, MA, USA) were coated by shaking them overnight at 4 °C with capturing goat anti-human TREM2 antibody (R&D Systems, Minneapolis, MN, USA) at a final concentration of 0.5 μg/ml in 50 mM carbonate–bicarbonate buffer (pH 9.6). The plates were then blocked with 2 % (w/v) bovine serum albumin in TTBS (20 mM Tris, 150 mM NaCl, pH 7.4 with 0.05 % (v/v) Tween-20) before sample incubation for 2 h at room temperature (RT). Samples were diluted (CSF 16×, THP-1 conditioned medium 16×, CHO-KI conditioned medium 50× and CHO-KI cell lysate 200×) in TTBS supplemented with 0.1 % (w/v) bovine serum albumin. The plates were then incubated with HRP-conjugated detecting mouse anti-human TREM2 antibody (Sino Biologics, Beijing, China) at a final concentration of 0.4 μg/ml for 1 h at RT. TTBS was applied for washing between each step. Finally TMB substrate (ANL produkter, Älvsjö, Sweden) was added for signal detection and development was stopped with 0.2 M H_2_SO_4_ (final concentration). The plates were analysed with a SpectraMAX 190 spectrophotometer at 450 nm (Molecular Devices, Palo Alto, CA, USA) and the data with SoftMax Pro software (Molecular Devices). Recombinant TREM2 was used as standard (Sino Biologics).

### CSF freeze–thaw cycles

CSF samples (*n* = 2) were subjected to five rounds of thawing and freezing. Briefly, samples of 15 μl were thawed for 90 s in a water bath at room temperature, incubated for 15 min on ice and then transferred to –80 °C for 1 h.

### T-tau, P-tau and Aβ42 ELISAs

CSF levels of T-tau, P-tau and Aβ42 were quantified with commercially available ELISAs; Innotest® hTau Ag, Innotest® phoshoTau (181P) [26, 27] and Innotest® β-amyloid 1–42 [28] (Fujirebio Europe, Gent, Belgium). Aβ1–38 (Aβ38), Aβ1–40 (Aβ40) and Aβ42 were analysed using a MSD Multi-Spot Assay System (Meso Scale Discovery, Rockville, MA, USA) with 6E10 (BioLegend, San Diego, CA, USA) as the detection antibody. The Meso Scale Discovery analyses, which were applied to a subset of patients in the Norwegian cohort, were carried out according to the manufacturers’ procedures.

### Statistical analyses

The statistical analyses were carried out with the Statistical Package for Social Sciences (SPSS, version 22; IBM, Armonk, NY, USA). Since the majority of the data were skewed, correlations were assessed by non-parametric Spearman rho and differences between groups by Mann–Whitney *U* test. Standardized residuals in multiple linear regression analyses gave no indication of violation of the normal distribution. All *p* values are two-tailed, since all hypotheses tested were two-sided. The significance level was set at 0.05. Graphs were created with GraphPad Prism (version 5.02; Graph Pad Software, La Jolla, CA, USA) or Statistica software (version 10; Statsoft, Uppsala, Sweden).

## Results

### Sensitive and specific detection of human TREM2 with a new sandwich ELISA

**Fig. 1:**
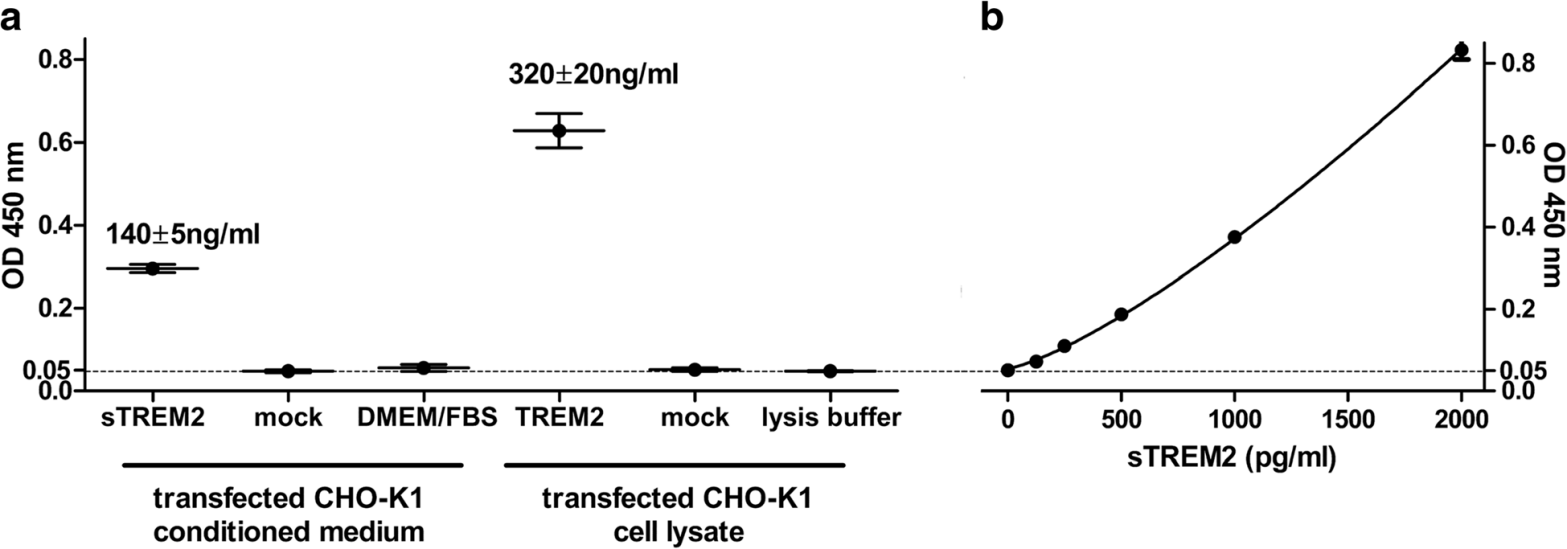
TREM2 ELISA. **a** Estimation of sTREM2 in conditioned medium and TREM2 levels in cell lysate of transiently transfected CHO-K1 cells. The readings of the negative control samples, mock-transfected cell lysate and medium were equal to or slightly below the zero point in the standard curve (OD ≈ 0.05; *n* = 2–4). **b** A representative standard curve in the ELISA assay (*n* = 2 at each concentration). Diluted CSF samples were read at OD values in the range of 0.2–0.8. *OD* optical density, *sTREM2* soluble triggering receptor expressed on myeloid cells 2

**Fig. 2:**
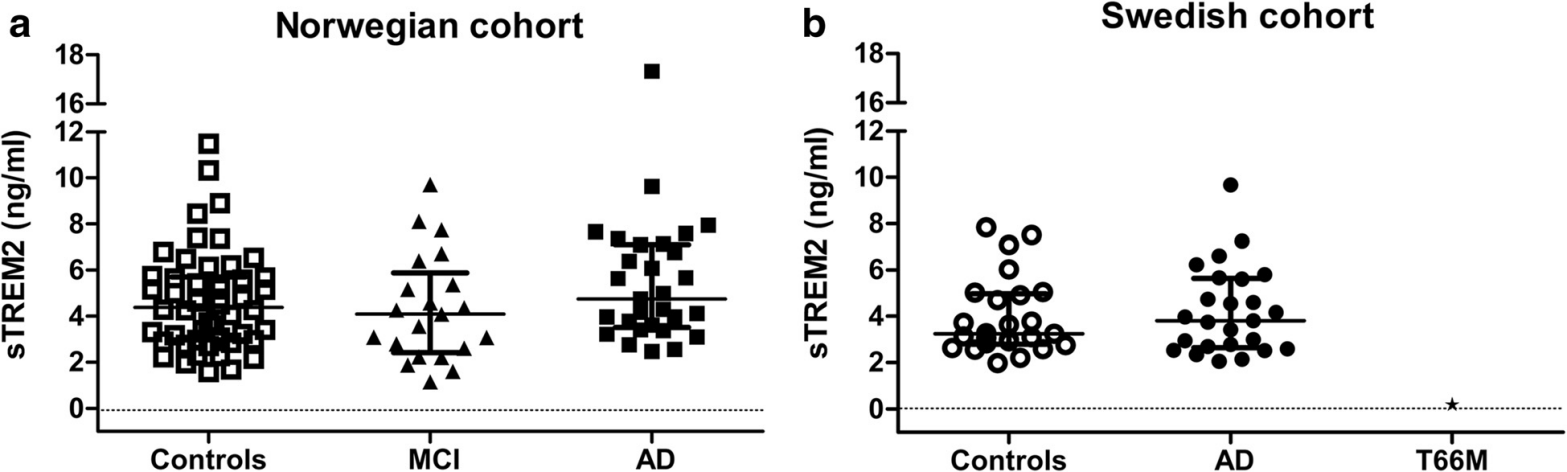
CSF sTREM2 levels did not depend on clinical diagnosis in two independent cohorts. **a** In the Norwegian cohort, sTREM2 levels did not significantly differ between controls (*n* = 50) and patients with Alzheimer’s disease (*AD*, *n* = 29) or mild cognitive impairment (*MCI*, *n* = 21). **b** The outcome was the same in the Swedish cohort after analysing AD patients (*n* = 25) and controls (*n* = 25). CSF of a single patient homozygous for the [p. T66M] mutation in *TREM2* was used as a negative control in the ELISA assay. *Smaller lines* and *larger lines* represent interquartile range and median, respectively. *sTREM2* soluble triggering receptor expressed on myeloid cells 2

Human TREM2 cells and mock-transfected CHO-K1 cells served as positive and negative control respectively for ELISA validation. The ELISA easily detected TREM2 in the cell lysate, as well as sTREM2 in the corresponding conditioned medium from TREM2-transfected CHO-K1 cells. The signals from conditioned medium and cell lysate of mock-transfected cells were at the level of the zero standard. Cellular TREM2 and released sTREM2 were thus detectable in complex biological samples without significant cross-reactivity (Fig. 1). Intra-day and inter-day variability was 6.7 ± 1.7 % and 5.7 ± 0.3 % respectively. The readings did not depend on dilution of the CSF samples (Additional file 1: Figure S1). The [p. T66M] mutation in *TREM2* markedly reduced cell surface transport and shedding of TREM2 [18]. A CSF sample from a patient homozygous for *TREM2* [p. T66M] was used to further ensure that the ELISA signal was solely due to sTREM2 in CSF. This CSF sample gave an ELISA signal very close to the zero standard (Fig. 2b) and below the estimated ELISA detection limit (62.5 pg/ml, Fig. 1b). Repeated freeze–thaw cycles did not affect CSF sTREM2 levels in two random CSF samples (Additional file 1: Figure S2).

### CSF sTREM2 levels did not relate to clinical diagnosis or degree of dementia

CSF sTREM2 was measured in samples from a Swedish AD/control cohort and a Norwegian AD/MCI/control cohort. The Norwegian cohort (*n* = 100) included controls (*n* = 50), MCI patients (*n* = 21) and AD patients (*n* = 29), and the Swedish cohort (*n* = 50) included controls (*n* = 25) and AD patients (*n* = 25). In line with the diagnosis, the AD patients had lower Mini-Mental State Examination (MMSE) scores and markedly reduced level of Aβ42 and elevated levels of T-tau and P-tau in CSF as compared with controls in both cohorts. The more pronounced separation in CSF Aβ42 levels between AD patients and controls in the Norwegian cohort was predicted because this cohort was diagnosed according to the revised NIA-AA criteria and controls were excluded if CSF Aβ42 was below 550 pg/ml. The groups in the Norwegian cohort were age matched, but the age range was wider among the controls. In the Swedish cohort, the controls were younger than the AD patients. The gender distribution was comparable in the control and MCI/AD groups of the Norwegian cohort. In the Swedish cohort, there were more women in both the control group and the AD group. The apolipoprotein E (ApoE) genotypes were similarly distributed in both cohorts, with more E3/E3 carriers in the control groups and more E3/E4 and E4/E4 carriers in the disease groups (Table 1).

There were no statistically significant differences in the level of sTREM2 between the MCI, AD and control groups in the Norwegian cohort (Fig. 2a and Table 1). The lack of difference in CSF sTREM2 levels between AD patients and controls was confirmed in the Swedish cohort (Fig. 2b and Table 1). All study groups displayed large variability (interquartile range/(median × 2) ≈ 30–40 %) but sTREM2 levels tended to be slightly elevated in the AD group in both cohorts. When the cohorts were merged to increase statistical power, there was still no difference in sTREM2 levels (*p* = 0.29) between AD patients (*n* = 54) and controls (*n* = 75). The level of sTREM2 and cognitive function (MMSE score) did not correlate even when the AD groups were merged (Spearman rho = 0.14, *p* = 0.33, *n* = 54). The lack of correlation between sTREM2 and MMSE score suggests that sTREM2 does not relate to cognitive ability in AD.

### CSF sTREM2 level correlated with age among controls

**Fig. 3:**
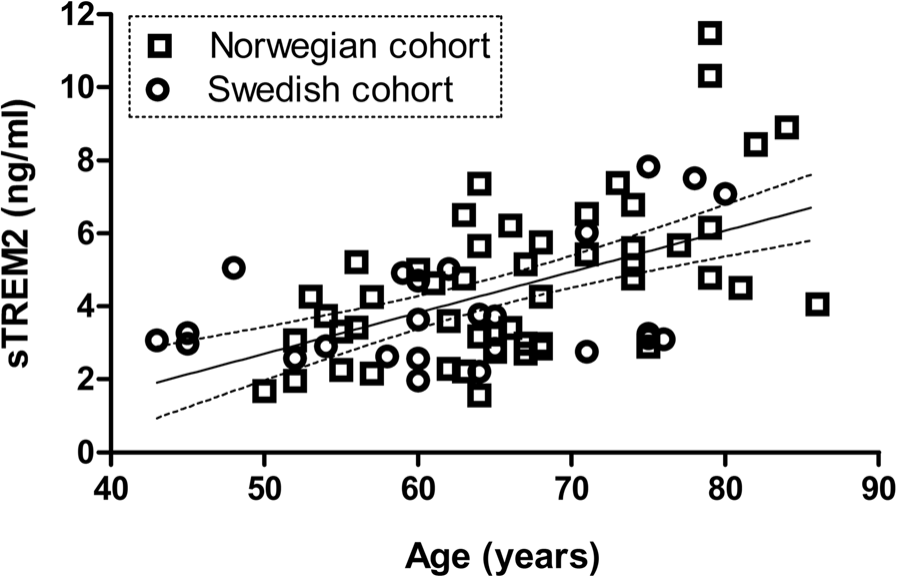
CSF sTREM2 levels correlated with age in the control groups. We found a significant positive correlation between age and sTREM2 in the control groups (Spearman rho = 0.50; *p* < 0.001; *n* = 75). The regression line (*dotted lines* representing the 95 % confidence interval) is based on merged data for both cohorts. *sTREM2* soluble triggering receptor expressed on myeloid cells 2

Next, because age is the main risk factor for AD, we questioned whether CSF sTREM2 levels related to normal aging. The analysis therefore focused on the control groups. As samples were assayed for sTREM2 in the same laboratory, the two cohorts were merged for this analysis. We found sTREM2 levels to increase with age in the control group, as demonstrated by a significant positive correlation (Spearman rho = 0.50; *p* < 0.001; *n* = 75; Fig. 3). Interpolation of linear regression showed that sTREM2 was ≈ 2.7 ng/ml at 50 years of age and ≈ 7.2 ng/ml at 90 years of age (i.e. almost a threefold increase). When cohorts were analysed separately, age and sTREM2 levels correlated significantly in the Norwegian cohort (Spearman rho = 0.53; *p* < 0.001; *n* = 50), but the correlation did not reach statistical significance in the Swedish cohort (Spearman rho = 0.35; *p* = 0.08; *n* = 25). In the AD groups we only found a positive correlation in the Swedish cohort (Spearman rho = 0.46; *p* = 0.02; *n* = 25). The correlation was weaker, but still positive, when the AD/MCI and control groups from both cohorts were merged (Spearman rho = 0.29; *p* < 0.001; *n* = 150; Table 2).

**Table 2:** Correlation analyses of CSF sTREM2 in relation to age in the Norwegian and the Swedish cohorts

	Correlations between sTREM2 and age										
	Norwegian cohort					Swedish cohort			Norwegian and Swedish cohort		
	All (*n* = 100)	AD (*n* = 29)	MCI (*n* = 21)	Control (*n* = 50)	Aβ42 > 700 (*n* = 46)	All (*n* = 50)	AD (*n* = 25)	Control (*n* = 25)	All (*n* = 150)	AD (*n* = 54)	Control (*n* = 75)
Age	*r* = 0.34	*r* = 0.08	*r* = 0.03	*r* = 0.53	*r* = 0.56	*r* = 0.35	*r* = 0.46	*r* = 0.35	*r* = 0.29	*r* = 0.00	*r* = 0.50
	*p* < 0.001	*p* = 0.69	*p* = 0.86	*p* < 0.001	*p* < 0.001	*p* = 0.01	*p* = 0.02	*p* = 0.08	*p* < 0.001	*p* = 1.00	*p* < 0.001

Correlation analyses of CSF sTREM2 in relation to age of AD, MCI and control patients of the two cohorts

Correlations are presented as Spearman rho (r) since most data not were normally distributed

*Aβ42* amyloid beta 1–42, *AD* Alzheimer’s disease, *CSF* cerebrospinal fluid, *MCI* mild cognitive impairment, *sTREM2* soluble triggering receptor expressed on myeloid cells 2

### Relation between sTREM2 levels and AD CSF biomarker levels, Aβ42 and P-tau/T-tau

AD is a complex and pathologically rather heterogeneous disorder with substantial individual variability in the tolerance to neuropathological changes. We hypothesized that the levels of TREM2 would reflect pathogenic processes rather than clinical symptoms. Therefore, we examined the relation between sTREM2 levels and the AD CSF biomarkers Aβ42, T-tau and P-tau in the larger Norwegian cohort in which the patients had been diagnosed according to the NIA-AA criteria.

**Fig. 4:**
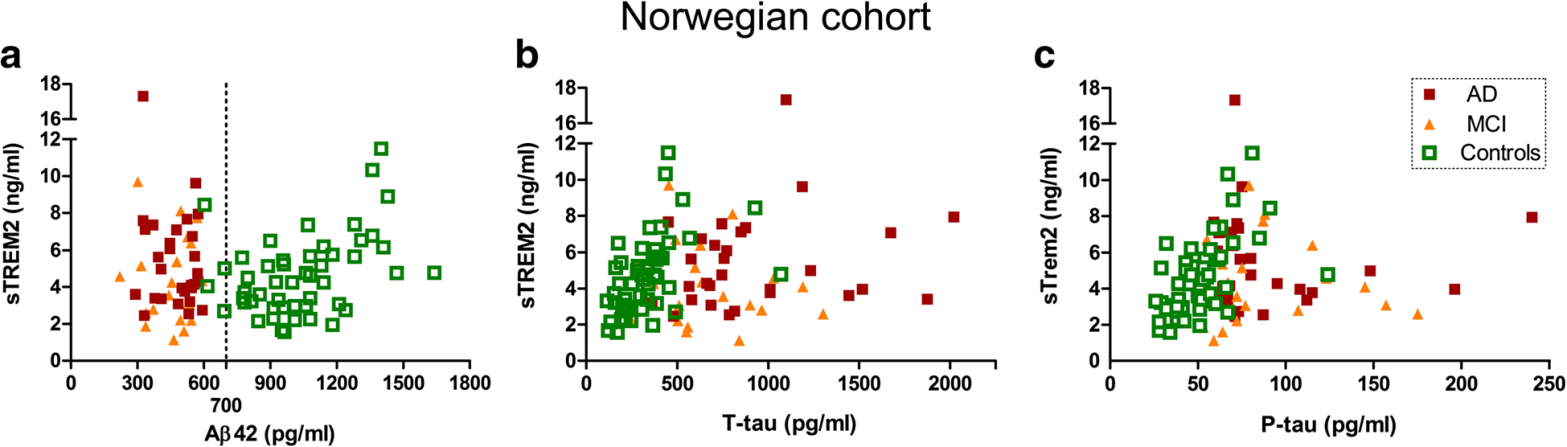
Relationship between sTREM2 levels and AD core biomarkers in CSF. The relationship between **a** sTREM2 and Aβ42, **b** sTREM2 and T-tau, and **c** sTREM2 and P-tau in CSF among all subjects in the Norwegian cohort. *Aβ42* amyloid beta 1–42, *AD* Alzheimer’s disease, *MCI* mild cognitive impairment, *P-tau* phosphorylated Tau, *sTREM2* soluble triggering receptor expressed on myeloid cells 2, *T-tau* total Tau

**Fig. 5:**
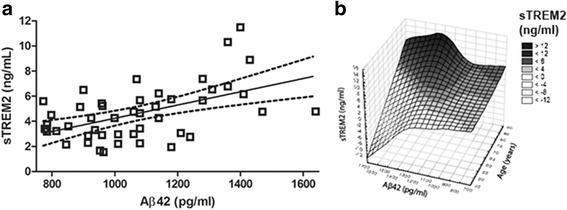
Relationship between sTREM2 and Aβ42 and age in subjects with high CSF Aβ42. Control subjects with high CSF Aβ42 (Aβ42 > 700 ng/ml, *n* = 46) were further analysed. **a** Relationship between sTREM2 and Aβ42 levels in CSF (Spearman rho = 0.44; *p* = 0.002; *n* = 46), the solid line representing the linear regression line (dotted lines the 95 % confidence interval). **b** Surface plot showing the relation between age (*z* axis), Aβ42 (*x* axis) and sTREM2 levels (*y* axis). *Aβ42* amyloid beta 1–42, *sTREM2* soluble triggering receptor expressed on myeloid cells 2

Aβ42 displayed a biphasic association pattern with sTREM2, with a distinction between AD/MCI and controls (Fig. 4a). There was a positive correlation between sTREM2 and Aβ42 among all controls (Spearman rho = 0.35; *p* = 0.01; *n* = 50) but not among AD/MCI patients (Table 3). Since Aβ42 and sTREM2 levels seemed to depend on the diagnostic state, the material was stratified and further examined. Controls with high Aβ42 (>700 pg/ml) were further analysed, since those with a lower Aβ42 (<700 pg/ml) might already have amyloid pathology in the brain. The cut-off value of 700 pg/ml was supported by comparison between CSF Aβ42 levels and amyloid-PET imaging in a separate group of Norwegian patients (Additional file 1: Figure S3). The correlation was stronger between CSF sTREM2 and Aβ42 among controls with high Aβ42 (>700 pg/ml; Spearman rho = 0.44; *p* = 0.002; *n* = 46; Fig. 5a and Table 3). CSF from many controls was also assayed with Meso Scale Discovery (MSD) for Aβ38, Aβ40 and Aβ42. In controls with high Aβ42 (>700 pg/ml) there were significant positive correlations between sTREM2 and Aβ38 (Spearman rho = 0.51; *p* = 0.004; *n* = 31), between sTREM2 and Aβ40 (Spearman rho = 0.53; *p* = 0.002; *n* = 31) and between sTREM2 and MSD estimates of Aβ42 (Spearman rho = 0.40; p = 0.03; *n* = 31) (Additional file 1: Figure S4 and Table 3). Estimation of Aβ42 with two independent techniques, Innotest® and MSD correlated strongly (Spearman rho = 0.88; *p* < 0.001; *n* = 38). MSD estimates of Aβ38 and Aβ40 in the same CSF sample also correlated very well (Spearman rho = 0.96; *p* < 0.001; *n* = 38), while correlations between MSD analyses of Aβ42 and Aβ38 or Aβ40 in the same samples were somewhat weaker (Spearman rho = 0.74; *p* < 0.001; *n* = 38) (Additional file 1: Figure S5 and Table S1).

**Table 3:** Correlation analyses of CSF sTREM2 in relation to other AD CSF biomarkers in the Norwegian and Swedish cohorts

	sTREM2 correlation							
	Norwegian cohort					Swedish cohort		
	All (*n* = 100)	AD (*n* = 29)	MCI (*n* = 21)	Control (*n* = 50)	Aβ42 > 700 (*n* = 46)	All (*n* = 50)	AD (*n* = 25)	Control (*n* = 25)
T-tau	*r* = 0.28	*r* = 0.22	*r* = –0.06	*r* = 0.57	*r* = 0.63	*r* = 0.17	*r* = 0.05	*r* = 0.29
	*p* = 0.005	*p* = 0.25	*p* = 0.81	*p* < 0.001	*p* < 0.001	*p* = 0.24	*p* = 0.82	*p* = 0.16
P-tau	*r* = 0.33	*r* = –0.04	*r* = 0.27	*r* = 0.63	*r* = 0.69	*r* = 0.17	*r* = 0.00	*r* = 0.44
	*p* = 0.001	*p* = 0.85	*p* = 0.24	*p* < 0.001	*p* < 0.001	*p* = 0.23	*p* = 0.99	*p* = 0.02
Aβ42	*r* = 0.04	*r* = –0.05	*r* = 0.07	*r* = 0.35	*r* = 0.44	*r* = 0.09	*r* = 0.35	*r* = 0.06
	*p* = 0.67	*p* = 0.79	*p* = 0.77	*p* = 0.01	*p* = 0.002	*p* = 0.55	*p* = 0.09	*p* = 0.79
				*n* = 32	*n* = 31			
Aβ38 (MSD)				*r* = 0.49	*r* = 0.51			
				*p* = 0.005	*p* = 0.004			
Aβ40 (MSD)				*r* = 0.51	*r* = 0.53			
				*p* = 0.003	*p* = 0.002			
Aβ42 (MSD)				*r* = 0.37	*r* = 0.40			
				*p* = 0.04	*p* = 0.03			

Correlations between CSF sTREM2 levels and levels of T-tau, P-tau, Aβ42, Aβ38 MSD, Aβ40 MSD and Aβ42 MSD in AD, MCI and control patients of the two cohorts. Correlations are presented as Spearman rho (r) since most data not were normally distributed

*Aβ42* amyloid beta 1–42, *AD* Alzheimer’s disease, *CSF* cerebrospinal fluid, *MCI* mild cognitive impairment, *MSD* Meso Scale Discovery, *P-tau* phosphorylated Tau, *sTREM2* soluble triggering receptor expressed on myeloid cells 2, *T-tau* total Tau

CSF sTREM2 correlated positively with the neurodegenerative biomarkers T-tau (Spearman rho = 0.57; *p* < 0.001; *n* = 50; Fig. 4b) and P-tau in the control group alone (Spearman rho = 0.63; *p* < 0.001; *n* = 50; Fig. 4c) and also when the control group was merged with the AD/MCI group. In the AD/MCI groups, the neurodegenerative biomarkers T-tau and P-tau did not relate to sTREM2. Similar plots and statistical calculations on data from the Swedish cohort only resulted in a positive correlation between sTREM2 and P-tau in controls (Spearman rho =0.44; *p* = 0.02; *n* = 25; Additional file 1: Figure S6 and Table 3).

sTREM2 levels correlated with age, Aβ42, Aβ40, Aβ38, T-tau and P-tau among controls with low likelihood of amyloid plaques, (Aβ42 > 700 pg/ml). Multiple linear regression was therefore performed using age and Aβ42 (Fig. 5a), Aβ40 or Aβ38 as the explanatory variable for sTREM2 levels. The level of sTREM2 associated positively with age (β1 = 0.12; *p* < 0.001) and Aβ42 (Innotest®) (β2 = 0.003, *p* = 0.01, *n* = 46; Fig. 5b and Additional file 1: Table S2). There were also significant positive associations between sTREM2 levels and age and MSD estimates of Aβ38 (age β1 = 0.08, *p* = 0.02; Aβ38 β2 = 0.001, *p* = 0.01, *n* = 31) and Aβ40 (age β1 = 0.07, *p* = 0.02; Aβ40 β2 = 0.0005, *p* = 0.01, *n* = 31; Additional file 1: Figure S7). The corresponding analysis of sTREM2, age and the neurodegenerative marker P-tau in the same cohort showed a significant association with both explanatory variables: age (β1 = 0.11, *p* = 0.003) and P-tau (β1 = 0.04, *p* = 0.02, *n* = 46). The same multiple linear regression analysis of the entire control group (Aβ42 Innotest®/T-tau/P-tau (*n* = 50) or Aβ38/Aβ40/Aβ42 MSD (*n* = 32)) gave close to identical results (Additional file 1: Table S2).

## Discussion

In the current study a new ELISA was developed and used to determine whether CSF sTREM2 related to AD, AD neurochemical biomarkers and cognitively healthy aging. A CSF sample from a patient homozygous for *TREM2* [p. T66M] and cultured cells with or without TREM2 expression were used to validate the ELISA. We examined whether sTREM2 levels related to diagnosis and the AD CSF biomarkers Aβ42, T-tau and P-tau, in two independent cohorts. While the Swedish cohort was diagnosed according to the NINCDS-ARDRA criteria [22], the Norwegian cohort was diagnosed according to the 2011 NIA-AA criteria with CSF biomarkers [2, 4]. Naturally there was a more prominent difference in the CSF AD biomarker signature between controls and AD/MCI in the Norwegian cohort. The levels of sTREM2, but not those of other biomarkers, were analysed in the same laboratory with a robust assay with low inter-plate and inter-day variability. We therefore considered it correct to pool the two cohorts when studying the relation between sTREM2 and aging. In contrast, each cohort was examined separately for correlations involving Aβ42, T-tau or P-tau since those data were generated in different laboratories. We did not find any significant differences in CSF the level of sTREM2 between AD patients, MCI patients and controls. However in the control group, the level of sTREM2 correlated positively with aging.

The innate immune receptor TREM2 is predominantly expressed in microglia and other myeloid cells [12, 29]. sTREM2 was first found in human CSF and conditioned medium of dendritic cells [19]. It can be generated by sequential proteolysis of membrane-bound TREM2 [20], or by an alternatively splicing pathway resulting in a transcript lacking the transmembrane domain [30]. sTREM2 levels in CSF could depend on the synthesis rate of membrane-bound receptors in TREM2-expressing cells, largely microglia and peripherally derived macrophages, transport to the cell surface, shedding and degradation of the sTREM2 fragment. TREM2 gene expression is regulated by factors inducing myeloid cell differentiation [31]. TREM2 receptor recycling and shedding can also be regulated by cytokines (e.g. interleukin-13) [32]. The turnover of sTREM2 is unknown but other fragments generated by shedding (e.g. soluble AβPP) have a short half-life (*t*_1/2_ ≈ 4 h) in the mouse brain [33].

We investigated whether sTREM2 levels altered with age, the most significant risk factor for AD and of importance to many neurodegenerative disorders. Aging is associated with gliosis, increased microglial activity [5] and astrocytosis in the brain [34]. In healthy controls we found a positive correlation between age and sTREM2 levels with almost a threefold increase from 50 to 90 years of age, suggesting that sTREM2 levels are related to the normal aging process. TREM2 mRNA was reported to increase by 50–100 % from 50 to 90 years of age with healthy human aging in several brain regions [12]. In AD brain, the level of TREM2 protein was found to increase roughly 50 % in the temporal cortex [35]. TREM2 mRNA expression was elevated to a similar extent and related to Braak staging [36]. Aging and AD thus enhanced TREM2 expression to a quantitatively similar extent in human brain. We found elevated CSF sTREM2 levels with aging but not with AD. Therefore mechanisms other than TREM2 expression presumably play a significant role in elevated sTREM2 levels with aging. We suggest that the age-dependent increased CSF sTREM2 levels partly reflect enhanced microglial TREM2 expression but also induced shedding and/or reduced clearance. In the AD/MCI groups, pathogenic processes including neurodegeneration might overshadow the effect of aging on CSF sTREM2. Our data demonstrate the importance of age-matched study groups.

sTREM2 has been suggested as a possible biomarker of neuroinflammation, which is increasingly being recognized as an early event in AD [37]. Therefore it is conceivable that the level of CSF sTREM2 is increased in early stages of AD, as it is in classical inflammatory conditions like multiple sclerosis [19]. However, the AD risk factor *TREM2* [p. R47H] and frontotemporal dementia mutations [25] are most probably loss of function, resulting in reduced TREM2 cell surface localization and shedding [18]. If such changes are relevant to sporadic AD one would instead predict decreased CSF sTREM2. Indeed, in a previous study CSF sTREM2 was reduced in AD patients as compared with controls although intra-group variability was extensive [18]. Our study also showed large intra-group variability but sTREM2 levels did not differ between AD and controls. Hence we could not confirm reduced CSF sTREM2 in AD; instead, levels of sTREM2 tended to be higher in AD than in controls in both cohorts. We also included a group of MCI patients to see whether there were any differences in sTREM2 relating to dementia. sTREM2 levels in MCI did not differ from either the control or the AD group. The different study outcome could depend on study populations or the assay format. Unlike the previous reports [18, 19], we measured absolute sTREM2 concentrations in the CSF samples. In the previous dementia study [18], in which CSF TREM2 was found to be reduced in AD, the AD patients were approximately a decade older than the controls. This is similar to the Swedish cohort in our report, but in contrast to the Norwegian cohort which was well matched for age. Age-matching thus does not seem to explain the differences in study outcome, since we found a positive correlation between sTREM2 levels and age.

The role of TREM2 in AD pathology is only partly clear. TREM2 may have a neuroprotective function by regulating microglial/macrophage polarization [38] and serving as a phagocytic receptor [18, 39, 40]. TREM2 could thus serve to clear soluble Aβ-aggregates and other toxic debris, and control the inflammatory reactions elicited by the early AD pathology. However, the effects of TREM2 deficiency on amyloid plaque load in AβPP transgenic mice are inconsistent [41–43]. We therefore compared the levels of sTREM2 with the AD neuropathological markers CSF Aβ42, T-tau and P-tau. Interestingly we found a positive correlation between CSF sTREM2 and T-tau, P-tau and Aβ42 in the control group. These correlations were not seen in the AD and MCI groups. The signature of low CSF Aβ42 and high CSF T-tau/P-tau is established and enables prodromal AD diagnostics [44]. Several studies have found the neurodegenerative markers T-tau and P-tau to increase almost threefold from 40 to 90 years with healthy aging [24, 45, 46]. Among aged subjects asymptomatic tauopathy is being reported [47]. Correlation of CSF sTREM2 levels with T-tau/P-tau could thus be an indirect effect of the age-dependent increase in T-tau/P-tau.

The positive correlation between sTREM2 and Aβ42 among controls is interesting because CSF Aβ42 is ultimately reduced in association with amyloid deposition in AD. CSF sTREM2 also correlated well with CSF measures of Aβ38 and Aβ40, and the different Aβ measures correlated well with each other. The increased CSF Aβ presumably reflects altered Aβ metabolism, and not Aβ42-selective changes associated with Aβ deposition. In a previous study CSF Aβ42 did not correlate with age among the cognitively healthy [24], while others reported a relation that best fit curve models of increasing and culminating Aβ42 [48, 49] and Aβ40 levels [49]. In the absence of amyloid deposits, an increased CSF-Aβ peptide level probably reflects an imbalance between production and clearance of Aβ. There is evidence of decreased Aβ clearance in aged individuals with sporadic AD [50]. Several mechanisms including age-related changes to the vascular basement membrane and impaired drainage along the lymphatic drainage pathway are probably involved [51]. Amyloid formation depends on seeding; that is, the local concentration of Aβ monomers must reach a critical threshold in order for fibril formation to begin [52]. Indeed, CSF Aβ42 was shown to transiently increase by 20–30 % in three inbred AβPP-transgenic models before it declined when amyloid plaques emerged [53]. We speculate that the positive correlation between CSF Aβ peptides and CSF sTREM2 among controls reflects a very early pre-symptomatic stage of dementia. These findings are preliminary and need to be further examined in other cohorts and in familial AD.

## Conclusions

A new sensitive TREM2 ELISA was used to determine CSF sTREM2 levels in cognitively healthy controls, and in patients with AD and MCI. In the cognitively healthy, we found CSF sTREM2 levels to increase with age, the most important risk factor of AD. There was no statistically significant difference in sTREM2 levels between diagnostic states (healthy control, AD and MCI), but sTREM2 levels correlated positively with the AD neuropathological CSF biomarkers Aβ42 and T-tau and P-tau in controls. Age should thus be considered when analysing sTREM2 in CSF. We speculate that increased CSF sTREM2 levels may reflect a very early microglial response to increased soluble Aβ42 in pre-symptomatic AD brain.

## Additional file

Additional file 1: Figure S1 showing ELISA validation; Figure S2 showing freeze–thaw cycles; Figure S3 showing PET imaging versus CSF Aβ42 in patients; Figure S4 showing a scatter plot of CSF sTREM2 in relation to CSF levels of Aβ38 MSD (A), Aβ40 MSD (B) and Aβ42 MSD (C) in the Norwegian AD/MCI/control cohort; Figure S5 showing a scatter plot of different CSF Aβ measures with Innotest® and MSD; Figure S6 showing a scatter plot of CSF sTREM2 in relation to levels of Aβ42 (A), T-tau (B) and P-tau (C) in CSF in the Swedish AD/control cohort; Figure S7 showing a surface plot of CSF sTREM2 in relation to age and Aβ38 MSD (A) and in relation to age and Aβ40 MSD (B) in controls; Table S1 presenting correlations between different CSF Aβ measures (Aβ42 Innotest®, Aβ38 MSD, Aβ40 MSD and Aβ42 MSD); and Table S2 presenting correlation analyses between different CSF sTREM2 and age and biomarkers (Aβ42 Innotest®, Aβ38 MSD, Aβ40 MSD and Aβ42 MSD, T-tau and P-tau). (PDF 647 kb)

## Abbreviations

AD: Alzheimer’s disease
ADAM: a disintegrin and metalloproteinase
ApoE: apolipoprotein E
Aβ: amyloid beta
Aβ42: amyloid beta 1–42
AβPP: Aβ precursor protein
CHO: Chinese hamster ovary
CSF: cerebrospinal fluid
CT: computed tomography
DAP12: DNAX activating protein 12
ELISA: enzyme-linked immunosorbent assay
MCI: mild cognitive impairment
MMSE: Mini-Mental State Examination
MRI: magnetic resonance imaging
MSD: Meso Scale Discovery
NFT: neurofibrillary tangle
NIA-AA: National Institute on Aging–Alzheimer’s Association
NINCDS: National Institute of Neurological and Communicative Disorders and Stroke
PET: positron emission tomography
P-tau: phosphorylated Tau
RT: room temperature
sTREM2: soluble triggering receptor expressed on myeloid cells 2
TREM2: triggering receptor expressed on myeloid cells 2
T-tau: total Tau
