# Supplementary material for: Cerebrospinal fluid soluble TREM2 in aging and Alzheimer’s disease
Source: Alzheimers Res Ther. 2016 Apr 27;8:17. doi: 10.1186/s13195-016-0182-1 (PMC4848774; doi:10.1186/s13195-016-0182-1)
Supplement: Additional file 1: — Figure S1 showing ELISA validation; Figure S2 showing freeze–thaw cycles; Figure S3 showing PET imaging versus CSF Aβ42 in patients; Figure S4 showing a scatter plot of CSF sTREM2 in relation to CSF levels of Aβ38 MSD (A), Aβ40 MSD (B) and Aβ42 MSD (C) in the Norwegian AD/MCI/control cohort; Figure S5 showing a scatter plot of different CSF Aβ measures with Innotest® and MSD; Figure S6 showing a scatter plot of CSF sTREM2 in relation to levels of Aβ42 (A), T-tau (B) and P-tau (C) in CSF in the Swedish AD/control cohort; Figure S7 showing a surface plot of CSF sTREM2 in relation to age and Aβ38 MSD (A) and in relation to age and Aβ40 MSD (B) in controls; Table S1 presenting correlations between different CSF Aβ measures (Aβ42 Innotest®, Aβ38 MSD, Aβ40 MSD and Aβ42 MSD); and Table S2 presenting correlation analyses between different CSF sTREM2 and age and biomarkers (Aβ42 Innotest®, Aβ38 MSD, Aβ40 MSD and Aβ42 MSD, T-tau and P-tau). (PDF 647 kb) [file 13195_2016_182_MOESM1_ESM.pdf]

SUPPLEMENTARY FIGURES (Fig S)

Supplementary figure 1 (Fig S1)

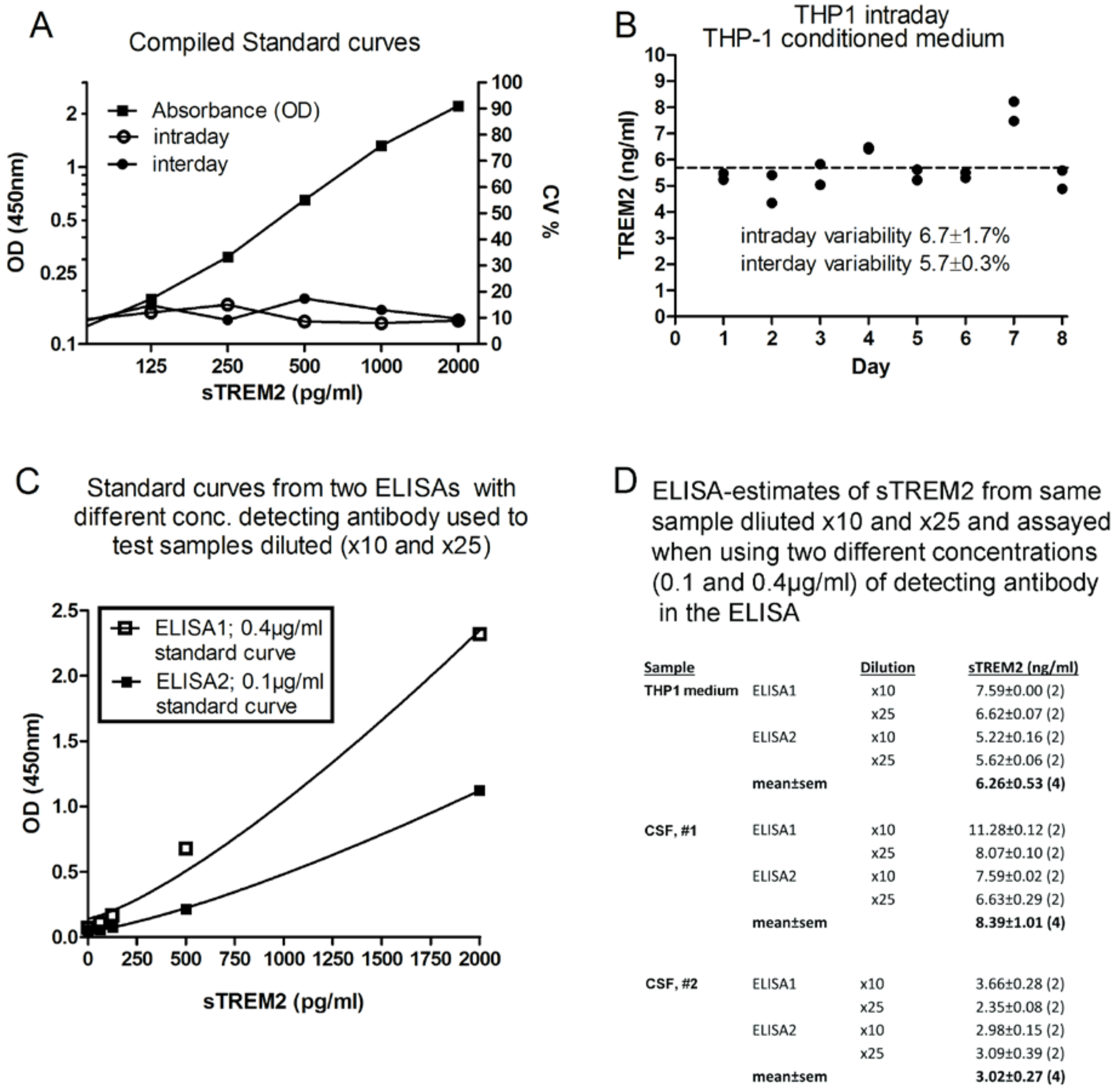

Sample		Dilution	sTREM2 (ng/ml)
**THP1 medium**	ELISA1	x10	7.59±0.00 (2)
		x25	6.62±0.07 (2)
	ELISA2	x10	5.22±0.16 (2)
		x25	5.62±0.06 (2)
	**mean±sem**		**6.26±0.53 (4)**
**CSF, #1**	ELISA1	x10	11.28±0.12 (2)
		x25	8.07±0.10 (2)
	ELISA2	x10	7.59±0.02 (2)
		x25	6.63±0.29 (2)
	**mean±sem**		**8.39±1.01 (4)**
**CSF, #2**	ELISA1	x10	3.66±0.28 (2)
		x25	2.35±0.08 (2)
	ELISA2	x10	2.98±0.15 (2)
		x25	3.09±0.39 (2)
	**mean±sem**		**3.02±0.27 (4)**

Fig S1 ELISA Validation (A) A compiled standard curve (n=6 from 3 different days) with variability. (B) Intraday and interday variability of a single sample conditioned medium (48h) of differentiated THP-1 cells. (C) Standard curves from ELISA with different concentration of the detecting antibody (0.1µg/ml and 0.4µg/ml). (D) Estimates of sTREM2-concentration in the same samples with two sample dilutions in these two ELISAs.

Supplementary figure 2 (Fig S2)

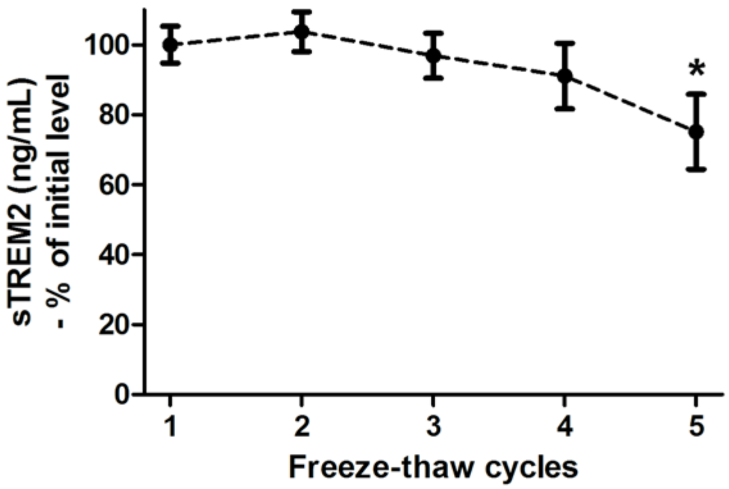

Fig S2 Freeze-thaw cycles Initial freeze-thaw cycles did not significantly affect sTREM2 levels. Two CSF samples were subjected to freeze-thaw cycles and sTREM2 levels were determined with sTREM2 ELISA. Data represent mean and standard deviation. Reduction in the sTREM2 level was analyzed by One-way ANOVA / Tukey's Multiple Comparison Test, * indicates significant reduction in sTREM2 level as compared to initial concentration (p=0.001).

Supplementary figure 3 (Fig S3)

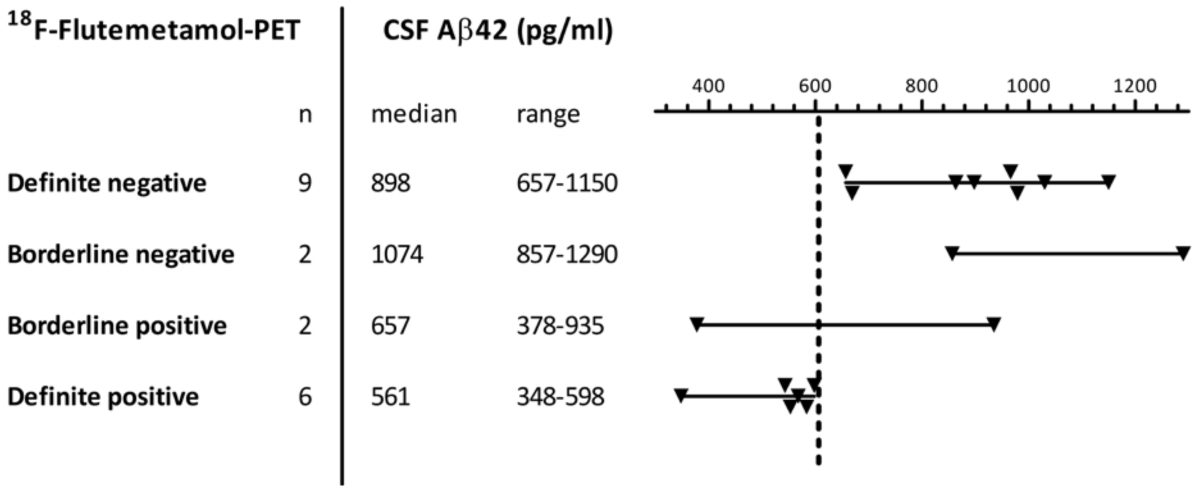

Fig. S3 **^{18}F-Flutemetamol-amyloid-PET imaging and cerebrospinal fluid Aβ42 level.** The results of ^{18}F-Flutemetamol-amyloid- positron emission tomography (PET) imaging (^{18}Flumetamol-PET) and CSF analyses of Aβ42 in 19 non-demented patients attending the Memory Clinic at Akershus University

Hospital. All patients with PET-scans interpreted as negative for brain amyloid, had CSF Aβ42 above 600 pg/ml (dotted line), while all with PET-scans interpreted as positive for brain amyloid had CSF Aβ42 below 600pg/ml. The only exception was one patient with CSF-Aβ42 935 pg/ml and a PET-scan read as marginally positive. Based on this, employing CSF Aβ42 > 700pg/ml as a cut-off would probably exclude most cases with substantial cerebral amyloid pathology.

Supplementary figure 4 (Fig S4)

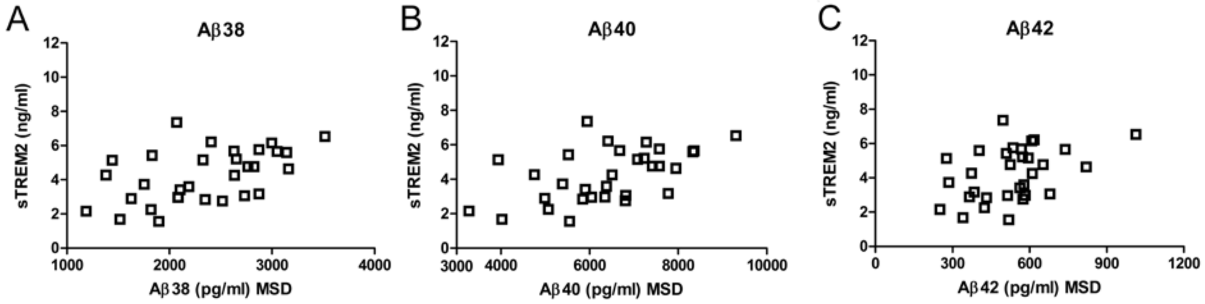

Fig S4. Relationship between CSF sTREM2 and Aβ38, Aβ40 and Aβ42 Meso Scale Discovery analyses

Relationship between cerebrospinal fluid sTREM2- and levels of (A) Aβ38, (B) Aβ40 and (C) Aβ42 measured with MSD Multi-Spot Assay System (Meso Scale Discovery) in a selection of controls in the Norwegian cohort with high CSF Aβ42 (>700 pg/ml; n=31).

Supplementary figure 5 (Fig S5)

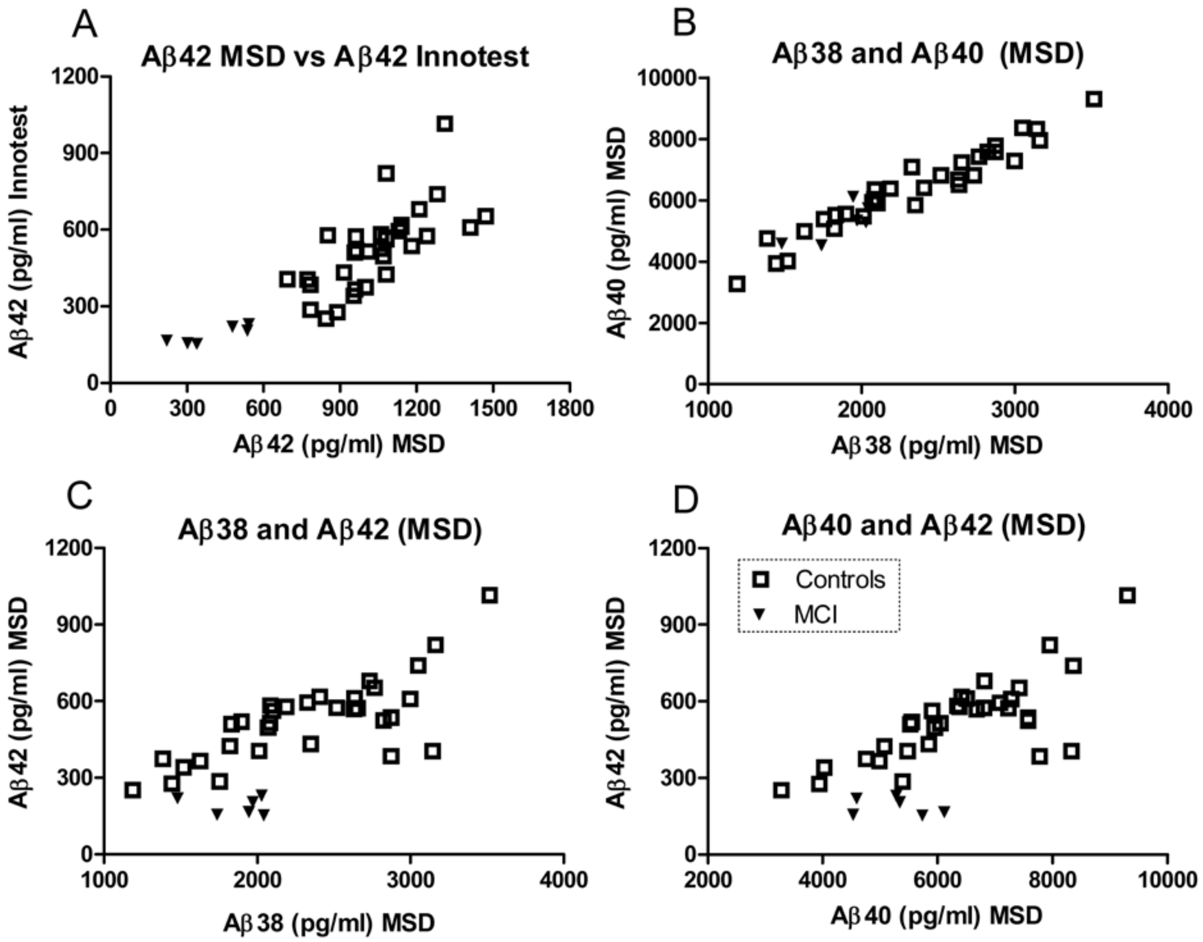

Fig S5. Relationship between Aβ-measures with MSD Multi-Spot Assay System (Meso Scale Discovery) and Aβ42 Innotest ELISA (A) Relationship between Aβ42-Innotest ELISA and Aβ42-MSD, (B) Aβ40-MSD and Aβ38-MSD, (C) Aβ42-MSD and Aβ38-MSD, (D) Aβ42-MSD and Aβ40-MSD in a selection of in the Norwegian cohort (n=38).

Supplementary figure 6 (Fig S6)

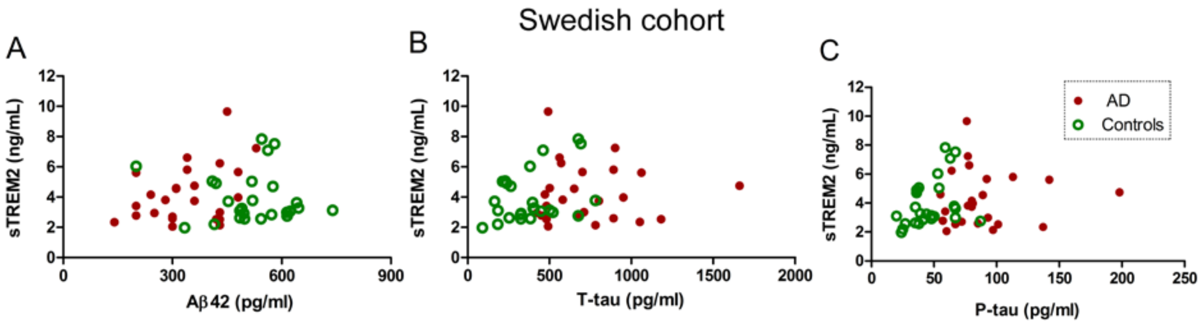

Fig S6. Relationship between CSF levels of sTREM2 and neurodegenerative markers in the Swedish cohort. Relationship between CSF sTREM2 levels and levels of (A) Aβ42, (B) T-tau and (C) P-tau in CSF among all subjects in the Swedish cohort.

Supplementary figure 7 (Fig S7)

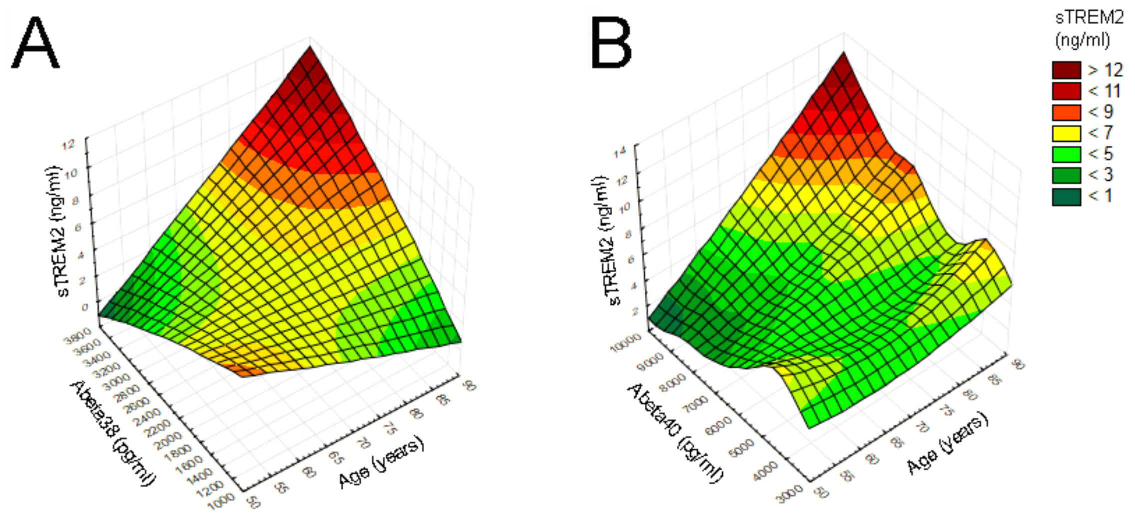

Fig S7. Relationship between cerebrospinal fluid soluble TREM2, age and Aβ38/Aβ40

Relationship between the CSF sTREM2 and age and (A) Aβ38, (B) Aβ40 among subjects with CSF level of Aβ42 >700pg/ml in the Norwegian cohort.

Supplementary Table 1 (Table S1)

Correlations between different Aβ peptide assays

Correlations between Aβ peptides (n=38)				
	Aβ42 Innotest	Aβ38 (MSD)	Aβ40 (MSD	Aβ42 (MSD)
Aβ42 Innotest		r=0.59 p<0.001	r=0.58 p<0.001	r=0.88 p<0.001
Aβ38 (MSD)	r=0.59 p<0.001		r=0.96 p<0.001	r=0.74 p<0.001
Aβ40 (MSD)	r=0.58 p<0.001	r=0.96 p<0.001		r=0.74 p<0.001
Aβ42 (MSD)	r=0.88 p<0.001	r=0.74 p<0.001	r=0.74 p<0.001	

Correlations are presented as Spearman Rho as not all data were normally distributed.

Supplementary Table 2 (Table S2)

Multiple linear regresion with CSF sTREM2

	All controls		Aβ42 (Innotest)>700pg/ml	
2nd explanatory variable	Age	2nd explanatory variable	Age	2nd explanatory variable
Aβ42 (n=50/46) Innotest	β1= 0.12 p<0.001	β2= 0.003 p=0.01	β1= 0.12 p<0.001	β2= 0.003 p=0.01
Aβ38 (n=32/31) (MSD)	β1= 0.07 p=0.02	β2= 0.001 p=0.01	β1= 0.08 p=0.02	β2= 0.001 p=0.01
Aβ40 (n=32/31) (MSD)	β1= 0.07 p=0.03	β2= 0.0005 p=0.01	β1= 0.07 p=0.02	β2= 0.0005 p=0.01
Aβ42 (n=32/31) (MSD)	β1= 0.08 p=0.01	β2=0.003 p =0.03	β1= 0.09 p=0.008	β2= 0.004 p=0.02
P-tau (n=50/46)	β1= 0.09 p=0.007	β2= 0.04 p=0.02	β1= 0.11 p=0.003	β2= 0.04 p =0.02
T-tau (n=50/46)	β1= 0.11 p=0.002	β2= 0.003 p=0.11	β1= 0.12 p=0.001	β2= 0.003 p=0.14

Aβ42 Innotest, P-tau and T-tau analyses: All controls n=50, Aβ42 (Innotest)>700pg/ml; n=46
Aβ38 (MSD), Aβ40 (MSD), Aβ42 (MSD) analyses: All controls n=32, Aβ42 (Innotest)>700pg/ml; n=31
